# Does Elderly Chronic Disease Hinder the Sustainability of Borderline Poor Families’ Wellbeing: An Investigation From Catastrophic Health Expenditure in China

**DOI:** 10.3389/ijph.2022.1605030

**Published:** 2022-08-25

**Authors:** Xiaocang Xu, Haoran Yang

**Affiliations:** ^1^ School of Economics and Management, Huzhou University, Huzhou, China; ^2^ Research Center for Economy of Upper Reaches of the Yangtse River, Chongqing Technology and Business University, Chongqing, China

**Keywords:** sustainable development, elderly chronic diseases, borderline poor families, catastrophic health expenditure, wellbeing

## Abstract

**Objectives:** Health and health expenditure caused by elderly chronic diseases are a global problem. As China has just lifted itself out of poverty in 2020, the sustainable development of Borderline Poor Families’ Wellbeing faces severe challenges. Therefore, it is of great practical significance to explore the impact of elderly chronic diseases on the catastrophic health expenditure of Borderline Poor Families.

**Methods:** Based on screening 8086 effective samples from China Health and Retirement Longitudinal Study (CHARLS) database and calculating catastrophic health expenditure, this paper uses two-part model and logit regression to discuss the impact of elderly chronic diseases on the sustainable development of Borderline Poor Families’ Wellbeing.

**Results:** The results showed that stroke, cancer, and liver disease caused the most catastrophic health expenditures and had the greatest impact on the Borderline Poor Families’ Wellbeing.

**Conclusion:** Therefore, in order to ensure the sustainable development of Borderline Poor Families’ Wellbeing, the government should strengthen the publicity of pre-prevention and post-healthcare of chronic diseases such as stroke, and combine pre-prevention policy with post-guarantee policy.

## Introduction

Healthcare expenditures due to population aging have become a global hot issue of concern to scholars [1]. According to the Seventh National Census (2021) in China, Proportion of people aged 65 and above in the total population rose from 10.5% in 2015 to 13.5% in 2020, which poses a serious challenge to both the government and families health expenditure burden. According to the WHO definition, when an individual’s health expenditure exceeds 40% of the family’s ability to pay, it means that the individual has incurred Catastrophic health expenditure currently, which well reflect the state of “poverty due to illness” of a family [2]. Such catastrophic health expenditure poses a serious challenge to the sustainability of Borderline Poor Families’ Wellbeing.

Chronic diseases in the elderly have a heavy economic burden on families, especially in countries with large elderly populations such as India and China (Kothavale et al., 2021 [3]; Hsieh and Qin, 2018 [4]). Puri and Pati (2022) explore the links between NCD prevalence, healthcare utilization, and expenditure in an indigenous older population of India [5]. Sun et al. (2020) [6] and Liu et al. (2020) [7] more specifically discussed the health expenditure of Depressive Symptoms among Rural Elderly in China. Swe et al. (2018) found a 4.6% increase in the inflation-adjusted costs of chronic diseases, including asthma, diabetes, heart disease, and parasitic diseases in Nepal [8]. This conclusion is further supported by research from scholars in developed countries (Meraya et al., 2015 [9]). After analyzing the trend of out-of-pocket medical expenses for chronic diseases in the United States over 10 years (1996–2005), Paez et al. (2009) found that per capita out-of-pocket medical expenses increased by 39.4% due to chronic diseases [10].

More seriously, the health economic burden caused by chronic diseases in the elderly has a greater impact on poor families (Deaton 2002 [11]; Aji et al., 2017 [12]; Somkotra and Lagrada, 2008 [13]; Counts and Skordis-Worrall [14]). In China, Xie H (2011) concluded that chronic diseases significantly increase medical expenses, and wealthy individuals are significantly more advantageous than poor patients with chronic conditions in resisting the impact of chronic diseases [15]. When the family health expenditure exceeds a certain threshold of family income and seriously affects the family’s wellbeing, it is called catastrophic health expenditure. It increases the risk of further poverty and further exacerbates inequality between the rich and the poor (Kang and Kim, 2018 [16]; Flores et al., 2008 [17]; Rezaei et al. 2019 [18]). Shumet et al. (2021) found that catastrophic health expenditure occurs in 64.2% of patients with chronic diseases, among which expensive medical services, transportation, and drugs are the reasons for catastrophic health expenditure in chronic patients in Northeast Ethiopia [19]. Kien et al. (2017) used principal component analysis (PCA) and concluded that household self-reported NCD diagnoses were most correlated with catastrophic health expenditure and poverty in northern Vietnam [20].

Therefore, the calculation of catastrophic health expenditure has become the primary work of relevant research (Kronenberg and Barros, 2014 [21]; Wagstaff et al., 2018 [22]). Global scholars took India, China, Iran and other developing countries as examples to calculate catastrophic health expenditure through family surveys over the years (Raban et al. 2013 [23]; Ma et al. 2019 [24]; Yazdi-Feyzabadi et al. 2018 [25]). For example, Fazaeli et al. (2015) used Bayesian logit approach discussed the main determinants of catastrophic health expenditure in Iranian households [26]. Cylus et al. (2018) calculated the catastrophic health expenditure in Europe through different calculation methods [27].

On the basis of calculating catastrophic health expenditure, different scholars have put forward different policy suggestions (Jung and Lee, 2022 [28]; Pak, 2021 [29]). First of all, some scholars believe that the fundamental source of health problems, such as environmental pollution, should be solved (Dimitris et al., 2020 [30]; Reza et al. 2019 [31]; Xu et al. 2022 [32]). Secondly, more scholars advocate the government’s medical system reform (Kheibari et al., 2019 [33]; Myint et al., 2019 [34]; Ahmadnezhad et al., 2019 [35]). For example, Tirgil et al. (2019) [36], Aryeetey et al. (2016) [37]and Karan et al. (2017) [38]discussed the impact of health insurance on out of Pocket Medical Expenditure of poor families in Turkey, Ghana and India. In addition, relevant studies taking China as a case have also begun to appear in recent years. For example, Guo et al. (2016) [39] and Zhou et al. (2022) [40] concluded that social pension expansion decreases medical costs in the Rural China. Wang andLi (2014) showed that the incidence and intensity of catastrophic health expenditure in elderly families with chronic disease were significantly affected by family-related factors and the medical treatment behavior of chronic disease patients [41]. Yu et al. (2019) concluded that medical insurance significantly increases the risk of catastrophic medical expenses for patients with chronic diseases, and this effect is more obvious in the lower-income group [42].

To sum up, there have been some studies on the causes of health expenditure such as environmental pollution, the calculation and corresponding policies of catastrophic health expenditure by global scholars. However, although there are more and more in-depth studies on the relationship between chronic diseases and health expenditures (Counts and Skordis, 2016) [14], few studies directly link chronic diseases with catastrophic health expenditures of Borderline poor families (Choi et al., 2015 [43]; Liu and Zhang. 2020 [44]). As a country with 190 million elderly people suffering from chronic diseases and having just completed poverty alleviation in 2020, China’s case is worth studying. Therefore, based on the latest data from China Health and Retirement Longitudinal Study (CHARLS) database, this paper examines the impact of different factors, especially chronic diseases in the elderly, on catastrophic health expenditures in China, and further explores the risk of returning to poverty brought about by their catastrophic health expenditures, which reflects the sustainability of Wellbeing for Borderline poor families. In addition, another innovation of this paper is to include seven of the most common and representative chronic diseases into the empirical analysis. While most existing studies discuss the relationship between chronic diseases and health spending by either integrating all chronic diseases or just one of them, this paper includes all seven of the most common and representative chronic diseases.

## Methods

### Conceptual Model: Catastrophic Health Expenditure Model

Catastrophic health expenditure is associated with a household’s out-of-pocket health expenditure, consumption expenditure, and survival expenditure (food expenditure), and is usually analyzed together with the mean intensity of catastrophic health expenditure. The mean intensity of catastrophic health expenditure reflects the impact of health expenditure on family living standards [45]. Relevant formulas and models are as Eqs 1–5:

$$\text{Catastrophic health expenditure}=\{\begin{array}{c} 1,\;\;\;\;\;\;\;\;\;\;if\;\;\;\frac{\;OOPE_{i}}{ctp_{i}}\geq 0.4\\ 0,\;\;\;\;\;\;\;\;\;\;\;if\;\;\frac{OOPE_{i}}{ctp_{i}}<0.4 \end{array}$$ (1)

$$ctp_{i}=exp_{i}-food_{i}\;\;$$ (2)

$$\text{Mean intensity of catastrophic health expenditure}=\{\begin{array}{c} OOPE_{i}/ctp_{i}-0.4\;,\;\;\;\;\;\;\;\;if\;\;\;\frac{\;OOPE_{i}}{ctp_{i}}\geq 0.4\\ 0,\;\;\;\;\;\;\;\;\;\;\;\;\;\;\;\;\;\;\;\;\;\;\;\;\;\;\;\;\;\;\;\;\;\;\;\;\;\;\;if\;\;\frac{OOPE_{i}}{ctp_{i}}<0.4 \end{array}$$ (3)

$$logit\;CHE=\beta_{1}+\beta_{1i}X_{i}+\varepsilon_{3i}$$ (4)

$$\text{INT}=\beta_{2}+\beta_{2i}X_{i}+\varepsilon_{4i}$$ (5)

Where, $OOPE_{i}$ refers to the out-of-pocket medical expenses of individual i ; $ctp_{i}$ refers to the affordability of individual i . It is the difference between the total consumption expenditure $\;exp_{i}$ and the expenditure for food purchase $food_{i}$ of individual i .It is also individual i 's non-food expenditure. $\beta_{1},\;\beta_{2}$ , $\beta_{1i}$ , $\beta_{2i}$ are regression coefficients. $\varepsilon_{3i}$ , $\varepsilon_{4i}$ are random disturbance terms. CHE is Catastrophic health expenditure, INT is Mean intensity of catastrophic health expenditure.

### Research Framework and Variable Selection

#### Research Framework

This paper takes Anderson model (The Behavioral Model of Health Services Use, BMHSU)as the research framework. Theoretical models of Health cost or medical expenditure have developed rapidly in the past 2 decades and the Behavioral Model of Health Services Use (BMHSU) proposed by Andersen (1995) is one of them. BMHSU is composed of three factors (including predisposing characteristics, enabling resources, and need) and their respective sub-variables, which are mainly used to explain individual medical and health service utilization behavior and its influencing factors. Andersen’s BMHSU model provides theoretical support and policy service evaluation framework for related research on medical and health service utilization. According to Anderson model, this paper selects the following relevant variables from CHARLS database.

#### Dependent Variables: Health Expenditure and Catastrophic Health Expenditure

Health expenditure and catastrophic health expenditure, respectively subdivided into two specific indicators, are used to represent the economic pressure of Borderline poor families. Health expenditure is measured by two specific indicators, namely, outpatient’s visit probability and the logarithmic form of health expenditure. Health expenditure take into account out-of-pocket expenditures for outpatient and hospitalization as well as healthcare expenditure. Catastrophic health expenditure is also measured by two specific indicators, namely, the occurrence probability and the mean intensity of catastrophic health expenditure. Among them, outpatient’s visit probability and the occurrence probability of catastrophic health expenditure are dichotomous variables, while the logarithmic form of health expenditure and the mean intensity of catastrophic health expenditure are continuous variables.

#### Core Independent Variables

To measure the effects of elderly chronic diseases on the catastrophic health expenditure of Borderline poor families, this paper selected the prevalence of seven chronic diseases, income status (or poverty status) and self-rated health status as core independent variables. The types of chronic diseases are referred to the studies of other scholars [20], and the most common or expensive chronic diseases are selected as representatives, which include Blood fat disease, Kidney disease, Stomach trouble, Chronic lung disease, Liver disease, Heart disease, and Stroke. Income status is divided into five levels, namely, Poverty, Close to the poverty, Low income above the poverty line, Middle-income, and High income. It is based on the poverty line (2,995), 10% above and 10% below the poverty line (2695.5; 3,294.5), the average income (6506.113) in 2018.

#### Control Variables

In this paper, the demographic characteristics (gender, age, marital status), and socioeconomic characteristics (education, medical insurance, endowment insurance) were selected as control variables.

### Empirical Method

**FIGURE 1:**
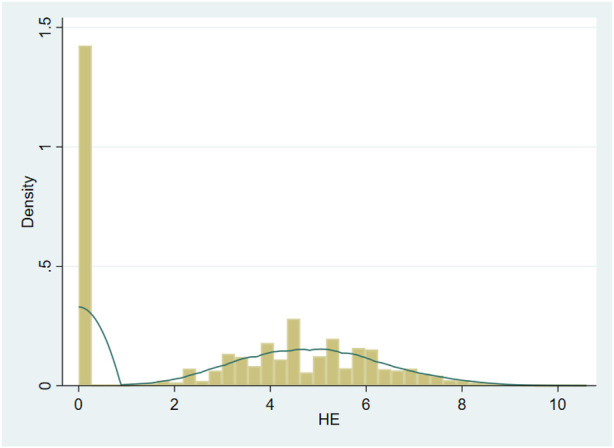
Data distribution of health expenditure (Huzhou, China, 2022). Source: China Health and Retirement Longitudinal Survey (CHARLS).

The health expenditure data used in this study presented a skewed distribution as shown in Figure 1. The vast majority of individuals had zero health expenditure in the past month; there is a significant gap in the amount of health expenditure between individuals who have health expenditure in the past month. Among them, the health expenditure of the investigated individual in the past month is 0, which may be in the following situations: First, the investigated individual is in good health in the past month and has no medical consumption demand, so the health expenditure in the past month is 0; Second, the respondents had health consumption demand in the past month, but subjectively chose to give up medical treatment due to economic pressure and accessibility of medical resources, resulting in 0 health expenditure in the past month. Combined with the above situation, for such skewness distribution data, if OLS regression is performed directly, it will often lead to selection bias and other endogenous problems.

Therefore, this paper uses the two-part model [46] to avoid the endogeneity of sample selection error. The two-part model is mainly used to deal with endogeneity problems caused by health expenditure with a large number of “0″ values. The two-part model includes the Outpatient’s visit probability model and the health expenditure level model, which are independent of each other, that is, the level of individual health expenditure has nothing to do with whether they choose medical treatment or not.

$$P_{i}\left(y_{i}>0|x_{i}\right)$$ (6)

$$E\left(y_{i}|x_{i},y_{i}>0\right)$$ (7)

$$E\left[\log\left(y_{i}\text{|}P_{i}>0\right)\right]$$ (8)

$$E\left(logy\text{|}x\right)=P_{r}\left(y>0\text{|}x\right)E\left(logy|y>0,x\right)$$ (9)

The first part of the two-part model usually uses probit model to estimate the probability of individuals choosing to use health services (Eq. 6). The second part estimates the level of individual health expenditure for individuals with health expenditure (Eqs 7–9).

### Data Source

The data used in this paper is from the China Health and Retirement Longitudinal Study (CHARLS), which was organized and implemented by The China Center for Social Sciences Investigation, Peking University. The survey was conducted among people aged 45 and older in a random sample of households. The database covers the life and health level of middle-aged and elderly statistics, with detailed social and economic data and high-quality physical and mental health status data. The most recent sample with complete data in 2018 was selected as the research object. The 2018CHARLS database contains the data of 19,816 respondents from 11,628 families, with a sufficient sample size. Based on this study, 8,086 samples were obtained after missing values were removed from the selected samples over 45 years old. The empirical operation was completed through Stata16.

## Results

### Descriptive Statistics

Table 1 is the descriptive statistics of this sample. The Outpatient’s visit probability was 29.1% on average, and the catastrophic health expenditure incidence was 27.2%. High blood pressure, heart disease and stroke account for the largest number of chronic diseases. 77.9% of the sample were poor. The average level of education is below secondary education, indicating that middle-aged and elderly group are generally not well educated. In addition, the majority of the samples were married, with an average age of 60.97. More women than men had medical insurance and endowment insurance.

**TABLE 1:** Variables and descriptive statistics (N = 8,086) (Huzhou, China, 2022).

Type	Variable	Variable description	Mean	Standard deviation	Minimum	Maximum
The dependent variable	Outpatient’s visit probability	Yes = 1; No = 0	0.166	0.372	0	1
	Logarithms of health expenditure	Continuous variable	2.981	2.649	0	10.60
	Whether catastrophic health expenditure occur	Yes = 1; No = 0	0.284	0.451	0	1
	Intensity of catastrophic health expenditure	Continuous variable	1.050	6.427	0	266.3
The independent variables	Chronic diseases	hypertension	0.071	0.256	0	1
		diabetes	0.036	0.187	0	1
		cancer	0.010	0.096	0	1
		Chronic lung disease	0.040	0.195	0	1
		Liver disease	0.024	0.152	0	1
		Heart disease	0.062	0.242	0	1
		stroke	0.048	0.213	0	1
	Income status	poverty	0.738	0.440	0	1
		Close to the poverty	0.017	0.131	0	1
		Low income above the poverty line	0.018	0.132	0	1
		middle-income	0.088	0.283	0	1
		High income	0.139	0.346	0	1
	Self -rated health	Scale from 1 to 5 (the lower the score, the better the health)	0.287	0.453	0	1
Control variables	Education	Not completing 9 years of compulsory education = 1; Completion of secondary education = 2; Completion of higher education = 3	1.307	0.488	1	3
	Age	Continuous variable	60.97	9.443	45	108
	Gender	Women = 0; Men = 1	0.455	0.498	0	1
	Marital status	Divorced, widowed, unmarried = 0; Have spouse = 1	0.867	0.340	0	1
	Endowment insurance	No = 0; Basic endowment insurance = 1; Land requisition endowment insurance = 2; Life insurance = 3	1.108	0.596	0	3
	Medical insurance	No = 0; Basic health insurance; Commercial medical insurance = 2	0.851	0.442	0	2

### Regression Results of Elderly Chronic Diseases on Health Expenditure

Table 2 shows the regression results of the relationship between elderly chronic diseases and health expenditure.

**TABLE 2:** Regression results of the two-part model (Huzhou, China, 2022).

Variable	Outpatient’s visit probability		Health expenditure	
	OR	Robust standard error	Coefficient	Robust standard error
Income status (control group: poverty group)				
Close to the poverty	0.965	0.194	0.145	0.193
Low income above the poverty line	1.374*	0.256	0.466**	0.223
Middle-income	1.000	0.079	−0.007	0.081
High income	1.212**	0.105	0.027	0.095
Self -rated health	2.870***	0.162	1.485***	0.067
Chronic diseases (control group: without chronic diseases)				
Hypertension	1.181*	0.120	0.479***	0.106
Diabetes	1.049	0.145	0.732***	0.150
Cancer	3.003***	0.755	1.279***	0.359
Chronic lung disease	2.379***	0.289	0.902***	0.148
Liver disease	2.275***	0.348	0.676***	0.205
Heart disease	1.572***	0.160	0.939***	0.121
Stroke	2.530***	0.289	1.244***	0.139
Medical insurance (control group: no medical insurance)				
Basic medical insurance	1.038	0.072	0.025	0.073
Commercial medical insurance	1.308*	0.209	0.232	0.165
Endowment insurance (control group: no endowment insurance)				
Basic endowment insurance	1.072	0.119	0.107	0.120
Endowment insurance for landless peasants	1.099	0.220	−0.033	0.224
Life insurance	1.127	0.170	0.355**	0.161
Other control variables				
Education	0.973	0.058	0.109*	0.062
Age	1.012***	0.003	0.007**	0.003
Gender	0.816***	0.045	−0.388***	0.058
Marital status	0.987	0.079	0.068	0.089
Constant	0.109***	0.031	1.717***	0.293
Observed value	8,086			

Note: ***p < 0.01; **p < 0.05; *p < 0.1.

#### Outpatient’s Visit Probability

First, the low-income group had the highest rate of medical visits, which was 1.4 times higher than the poor group (OR = 1.374). At the same time, the high income group was 1.2 times as high as the poor group (OR = 1.212), but the other income groups did not pass the significance test. Second, patients with chronic diseases are more likely to see a doctor than those without chronic diseases. In addition to diabetes, hypertension and other chronic diseases passed the significance test at *p* < 0.1 and *p* < 0.01 confidence levels, respectively, indicating that chronic diseases had a significant impact on the probability of seeing a doctor. Third, having medical insurance or endowment insurance increases the probability of seeing a doctor. Fourthly, the group with poor self-rated health status passed the test at *p* < 0.01 significance level, and the probability of seeing a doctor in the group with poor health was 2.9 times higher than that in the group with the best health status (OR = 2.870). Finally, age difference and gender difference also significantly affect the probability of medical treatment of residents, and the elderly population and female population are higher.

#### Logarithmic Form of Health Expenditure

The regression results in Table 2 indicate some information. For example, first, there are some differences in the influence mechanism of the probability of seeing a doctor and the health expenditure, which is more obvious in endowment insurance and education level. Those with pension insurance significantly reduced the health expenditure of residents compared to those without pension insurance (−0.033), while the health expenditure increased by 10.9% for each level of education. Second, the health expenditure of the low-income group increased significantly compared with the poor group (*p* < 0.05). Third, all seven chronic diseases increase the level of health expenditure to varying degrees, and the results are very significant. Cancer had the highest impact on health expenditure (1.279). Fourthly, health expenditure increases by 14.85 per cent (1.485) for every 1 increase in the self-rated health status score. Finally, the regression results also show that, except for marital status, individual demographic variables have a significant impact on health expenditure.

### Regression Results of Elderly Chronic Diseases on Catastrophic Health Expenditure

Table 3 shows the regression results of the relationship between elderly chronic diseases and catastrophic health expenditure.

**TABLE 3:** Regression results of Catastrophic health expenditures (Huzhou, China, 2022).

Variable	Catastrophic health expenditure		Mean intensit	
	OR	Robust standard error	Coefficient	Robust standard error
Income status (control group: poverty group)				
Close to the poverty	0.941	0.183	−0.225	0.323
Low income above the poverty line	1.505**	0.275	0.550	0.472
Middle-income	0.974	0.076	0.190	0.239
High income	0.862	0.082	−0.368***	0.126
Self -rated health	2.987***	0.169	1.098***	0.188
Chronic diseases (control group: without chronic diseases)				
Hypertension	1.224**	0.122	0.219	0.183
Diabetes	1.547***	0.199	0.239	0.279
Cancer	1.806**	0.454	3.419*	2.028
Chronic lung disease	1.872***	0.236	1.387**	0.556
Liver disease	1.814***	0.291	0.459	0.350
Heart disease	1.757***	0.183	0.585	0.443
Stroke	2.240***	0.260	0.914**	0.432
Medical insurance (control group: no medical insurance)				
Basic medical insurance	1.116	0.078	0.226	0.166
Commercial medical insurance	0.681**	0.126	−0.325	0.231
Endowment insurance (control group: no endowment insurance)				
Basic endowment insurance	0.936	0.104	−0.056	0.296
Endowment insurance for landless peasants	0.823	0.165	−0.549	0.355
Life insurance	0.908	0.140	−0.034	0.398
Other control variables				
Education	0.918	0.055	−0.009	0.128
Age	1.022***	0.003	0.023***	0.008
Gender	0.800***	0.044	−0.177	0.149
Marital status	1.603***	0.139	0.450**	0.214
Constant	0.049***	0.014	−1.259**	0.626
Observed value	8,086			

Note: ***p < 0.01; **p < 0.05; *p < 0.1.

#### Catastrophic Health Expenditure

First, income has different influences on catastrophic health expenditure. Catastrophic health expenditure was higher in the low-income group, 1.5(OR = 1.505) times that in the poorest group, and passed the test at the significance level of *p* < 0.05. Second, catastrophic health expenditure levels were significantly affected by all seven chronic diseases. Catastrophic health expenditure was significantly higher in patients with chronic disease than in those without chronic disease. Thirdly, medical insurance and endowment insurance have different influences on catastrophic health expenditure level. Among them, commercial medical insurance reduced the risk of catastrophic health expenditure occurrence (*p* < 0.05), and other medical insurance and endowment insurance did not pass the significance test. Fourth, the catastrophic health expenditure probability increased significantly by 3 times for each higher grade of self-rated health status score (OR = 2.987). Fifthly, the individual’s demographic characteristic variables except education level all passed the significance test at *p* < 0.01 level. The probability of catastrophic health expenditure increased by 1.02 times with each increase of age (OR = 1.022), and the probability of catastrophic health expenditure risk in males was lower than that in females (OR = 0.800). In addition, catastrophic health expenditure in spousal households were 1.6 times higher than those in single households (OR = 1.603).

#### Mean Intensity of Catastrophic Health Expenditure

First, the mean intensity of catastrophic health expenditure is significantly different from the influencing factors of catastrophic health expenditure. The mean intensity of catastrophic health expenditure was significantly affected by chronic disease, primarily cancer, chronic lung disease, and stroke alone. It shows that compared with other chronic diseases, these three chronic diseases have a deeper impact on residents’ living standards, and cancer is the one with the deepest impact. Second, the mean intensity of catastrophic health expenditure of the low- and middle- income groups increased with the increase of income level, indicating that the current income cannot cope with catastrophic health expenditure well and the risk resistance is weak. The mean intensity of catastrophic health expenditure of the high-income group decreased significantly (*p* < 0.01), and the mean intensity of catastrophic health expenditure of the individual decreased by 36.8% for every 1 unit increase in income. Third, the mean intensity of catastrophic health expenditure was reduced by other types of medical insurance and endowment insurance except basic medical insurance. Fourthly, the mean intensity of catastrophic health expenditure was significantly affected by self-rated health status (*p* < 0.01), and increased by 1.1 (1.098) for every 1 increase in the score. Finally, male, married and those with higher education level had lower the mean intensity of catastrophic health expenditure.

### Robustness Test: Change the Threshold of Catastrophic Health Expenditure

To test the regression results in Table 3, the robustness test was carried out (Table 4) by change the catastrophic health expenditure threshold in the catastrophic health expenditure model. The catastrophic health expenditure threshold is selected according to previous studies of scholars and replaced with two dimensions of 30% and 35%.

**TABLE 4:** Robustness tests for Catastrophic health expenditures (Huzhou, China, 2022).

Threshold value	Catastrophic health expenditure		Mean intensity	
Variable	30%	35%	30%	35%
	OR	OR	Coefficient	Coefficient
Income status (control group: poverty group)				
Close to the poverty	0.923	0.967	−0.226	−0.226
Low income above the poverty line	1.311	1.479**	0.558	0.554
Middle-income	1.005	1.004	0.190	0.190
High income	0.935	0.916	−0.370***	−0.369***
Self -rated health	2.978***	2.986***	1.121***	1.109***
Chronic diseases (control group: without chronic diseases)				
Hypertension	1.353***	1.297***	0.224	0.221
Diabetes	1.540***	1.568***	0.248	0.243
Cancer	1.806**	2.042***	3.433*	3.426*
Chronic lung disease	1.973***	1.903***	1.400**	1.393**
Liver disease	1.851***	1.848***	0.472	0.466
Heart disease	1.780***	1.798***	0.597	0.591
Stroke	2.350***	2.198***	0.932**	0.922**
Medical insurance (control group: No medical insurance)				
Basic medical insurance	1.104	1.114	0.228	0.227
Commercial medical insurance	0.798	0.701**	−0.329	−0.327
Endowment insurance (control group: No endowment insurance)				
Basic endowment insurance	0.966	0.988	−0.057	−0.057
Endowment insurance for landless peasants	0.837	0.836	−0.553	−0.551
Life insurance	0.978	0.991	−0.035	−0.035
Other control variables				
Education	0.959	0.942	-0.010	−0.009
Age	1.023***	1.022***	0.023***	0.023**
Gender	0.765***	0.777***	−0.182	−0.179
Marital status	1.520***	1.556***	0.458**	0.454**
Constant	0.056***	0.049***	−1.269**	−1.264**
Observed value	8,086			

Note: ***p < 0.01; **p < 0.05; *p < 0.1.

The influence of chronic disease on catastrophic health expenditure and its intensity was independent of the catastrophic health expenditure threshold. First of all, the conclusions in Table 4 are consistent with those in Table 3, all seven chronic diseases still passed the significance test at 30% and 35% thresholds (*p* < 0.05), proving that chronic diseases do increase the risk of catastrophic health expenditure in patients. Among the seven chronic diseases, stroke, chronic lung disease and liver disease were still the high-risk chronic diseases leading to catastrophic health expenditure regardless of the threshold. In addition, catastrophic health expenditure increased to varying degrees for all the seven chronic diseases, which had an impact on family living standard, among which cancer still had the greatest impact on family living standard (3.433, 3.426). The impact of residents’ self-rated health, age and marital status on catastrophic health expenditure is also basically consistent with the conclusion in Table 3, which significantly affects catastrophic health expenditure and its intensity, and has nothing to do with threshold value.

## Discussion

The ongoing COVID-19 pandemic poses a significant challenge to poverty alleviation and the sustainable development of future wellbeing for Borderline Poor Families in China, who are vulnerable due to the high health costs of chronic diseases. After selecting 8086 effective samples from CHARLS database and calculating catastrophic health expenditure, this paper used two-part model, that can solve the endogeneity problem, to discuss the impact of elderly chronic diseases on the sustainability of Borderline Poor Families’Wellbeing. Some interesting results were found.

Firstly, Chronic diseases have a significant impact on health expenditure. The regression results showed that most chronic diseases had a significant positive impact on health expenditure and catastrophic health expenditure incidence. Under the background of multiple chronic diseases being included in outpatient reimbursement, patients with chronic diseases tend to increase their health expenditure to cope with the impact of diseases on their bodies. However, the reimbursement ratio is often less than 100%. Patients with chronic diseases need long-term medication and hospitalization, which leads to increased out-of-pocket expenditures, consistent with the conclusions of previous scholars [15]. The chronic diseases that contributed most to increasing health expenditure were cancer, stroke, and liver disease.

Secondly, Individuals with chronic diseases have a higher incidence of returning to poverty than those without chronic diseases. Some scholars have proposed that individuals with catastrophic health expenditure are consistent with those in poverty [47, 48], and catastrophic health expenditure has a “pro-poverty” effect [44]. Therefore, catastrophic health expenditure individuals can be associated with their corresponding poverty status. In this regression, chronic diseases were significantly poor due to illness. Other scholars have also reached similar conclusions [7, 46]. The three chronic diseases that have the greatest impact on poverty continue to be stroke, cancer, and liver disease. Some scholars have found that cancer patients have the highest risk of fatal stroke [49]. For patients with chronic diseases, they may suffer from not just one chronic disease, but multiple chronic diseases, and the interaction of chronic diseases will cause higher harm to the body and higher health expenditure. As a result, patients with chronic diseases fell into poverty under the background of “income reduction” and “expenditure increase.”

Finally, Borderline poor families are more affected than other income groups. Regression results show that poor people have a low probability of medical treatment, resulting in less health expenditure. At the statistical level, it cannot be inferred that poor people are in better health or are not poor due to illness. Considering their own economic situation, poor people are likely to choose not to seek medical treatment even if they get sick, and there will be no health expenditure. The Borderline Poor Families above the poverty line have the highest rate of medical treatment, resulting in significantly the highest health expenditure. If the poor are the potential targets of poverty due to disease, then the low-income population is the observable high-risk group of poverty due to disease. In the regression results for catastrophic health expenditure incidence, the low-income population still passed the significance test. Overall, with the increase of income, health expenditure decreases, and the probability of catastrophic health expenditure also decreases, confirming other scholars’ conclusions [41, 50]. Although the income of low-income people is higher than that of poor people, catastrophic health expenditure poverty is not degraded, and the mean intensity is higher, indicating that chronic diseases have the deepest and most extensive impact on low-income people in the sample population, and the probability of returning to poverty due to disease is also higher.

The study has some policy implications for eliminating catastrophic health expenditure and ensuring the sustainability of the Borderline Poor Families’ wellbeing. For example, the government should combine pre-prevention policy and post-guarantee policy. On the one hand, pre-prevention at source is a low-cost and effective way to mitigate the impact of chronic diseases on health expenditures and the return to poverty caused by them. However, according to the empirical results, although buying health insurance plays a certain role, it does not significantly reduce and catastrophic health expenditures. It shows that the measures were taken after the event plays a limited role in improving poverty. Therefore, on the other hand, the prevention and publicity of chronic diseases should be strengthened, and the middle-aged and elderly should be encouraged to regularly test their physical indicators every year, which can improve post-guarantee capacity to prevent patients with chronic diseases from reducing their outpatient’s visit probability because they are worried about high medical costs. Ultimately, the sustainability of Borderline Poor Families’ wellbeing is a long-term challenge with a formidable task.

## Data Availability

The original contributions presented in the study are publicly available. This data can be found here: http://charls.pku.edu.cn/. Further inquiries can be directed to the corresponding authors.
